# The Global Burden and Temporal Trend of Kidney, Bladder, and Prostate Cancers Attributed to Smoking From 1991 to 2021

**DOI:** 10.7759/cureus.86389

**Published:** 2025-06-19

**Authors:** Zewei Liu, Xun Li

**Affiliations:** 1 Department of Urology, People’s Hospital of Xinjiang Uygur Autonomous Region, Urumqi, CHN

**Keywords:** bladder cancer, kidney cancer, prostate cancer, sdi, smoking

## Abstract

Background

Smoking remains a major risk factor for urological cancers, but its global burden across socio-demographic index (SDI) lacks quantification. Moreover, differences in age and gender among populations also have a significant impact on tobacco control, thereby influencing the incidence of urological cancers. Clarifying these trends is crucial for prioritizing targeted prevention policies.

Methods

The data were extracted from the Global Burden of Disease (GBD) 2021 platform. Patients were initially diagnosed using the International Classification of Diseases, 10th edition (ICD-10) for urological cancers and classified into smoking population according to the definition of GBD. The annual percent change (APC) and average annual percent change (AAPC) were measured by the Joinpoint regression program.

Results

Although age-standardized rates of DALYs (ASDRs) for urological cancers attributed to smoking showed a downward trend globally (-1.16, 95% confidence interval (CI): -1.29 to -1.04, -1.72, 95%CI: -1.86 to -1.59, and -1.84, 95%CI: -1.95 to -1.74), the number of disability-adjusted life years (DALYs) continued to increase from 1991 to 2021. In regions with high SDI, the highest numbers and age-standardized rates (ASRs) of DALYs were observed. Meanwhile, high and high-middle SDI regions experienced the most significant declines in ASDRs from 1991 to 2021. The burden of urological cancers for men was much higher than that for women. The number of DALYs showed a trend of younger aggregation as the SDI decreased, and the upward trend of ASDRs with age became gentler.

Conclusion

The current study analyzed the burden of kidney, bladder, and prostate cancers attributed to smoking around the world from 1991 to 2021, providing comprehensive information for the prevention of urological cancers and policymaking.

## Introduction

Kidney, bladder, and prostate cancers are among the most prevalent urologic tumors globally, imposing a significant oncological burden on individuals and societies [1]. According to GLOBOCAN 2020, the global incidence of kidney cancer was 431,288 people with a mortality of 179,368 people [2]. Bladder and prostate cancers rank ninth and second in incidence, and fifth and 13th in mortality [3]. These epidemiological patterns correlate with the socio-demographic index (SDI), but the underlying mechanisms, especially regarding smoking-attributable burdens, remain unclear.

Diagnostic and treatment advancements for urological cancers have been notable. However, in developing countries, the incidence and mortality of these cancers continue to rise, contrasting with trends in developed nations [4, 5]. In undeveloped regions, limited medical resources and inaccurate diagnosis lead to underreporting of incidence and mortality [5, 6]. Understanding the epidemiological characteristics of urological cancers across regions with varying SDI levels is crucial for effective disease prevention and policymaking.

Among the established risk factors for kidney, bladder, and prostate cancers, smoking is one of the most common [1, 7, 8]. Despite the prevalence of smoking decreasing in most parts of the world from 1990 to 2015 at 28.4% and 34.4% for men and women, respectively, the mortality rate caused by smoking still ranks fifth in the world. Moreover, the smoking rate has been continuously increasing in some low-SDI regions, such as Congo, Azerbaijan, and Kuwait [9]. Furthermore, the number of mortality and disability-adjusted life years (DALYs) showed an increased trend from 1990 to 2019 for kidney, bladder, and prostate cancers [10-12]. Despite this, a comprehensive, global-scale study analyzing these three urological cancers attributed to smoking is lacking.

Therefore, this study aims to systematically analyze the temporal and spatial distribution of years lived with disability (YLDs), years of life lost (YLLs), and DALYs for kidney, bladder, and prostate cancers attributable to smoking using the Global Burden of Disease Study platform 2021 (GBD 2021). The analysis will be conducted across different ages, genders, and SDI levels, filling a critical gap in current research.

## Materials and methods

Data sources

The data used in the study was extracted from GBD 2021 (https://vizhub.healthdata.org/gbd-results). Extraction parameters included selecting the period from 1991 to 2021, causes of kidney cancer, bladder cancer, and prostate cancer, smoking as the risk factor, and stratification by age (five-year bands), gender, and SDI. The data from GBD 2021 were systematically compiled through integrative processing of diverse global data sources, including but not limited to the following: published literature, vital statistics registries, population surveys, census records, administrative databases, disease registries, primary research studies, institutional reports, and satellite remote-sensing imagery. After processing through disease burden estimation and risk factor attribution models, the results generated comprehensive estimates of health burdens and associated risk factors, stratified by age and sex, for 371 diseases and injuries and 87 risk factors across 204 countries and territories and 811 subnational regions [13]. The present study analyzed the spatial and temporal trends and age-standardized rates (ASRs) of YLDs, YLLs, and DALYs for kidney, bladder, and prostate cancers from 1990 to 2021. The data included disparities by age, gender, and SDI (high, high-middle, middle, low-middle and low SDI).

Definition of urological cancers

The International Classification of Diseases, 10th edition (ICD-10) used for identifying urological cancers in GBD 2021 included C64-C64.2, C64.4-C64.6, C64.8-C64.9, C65-C65.2, C65.9, D30.0-D30.1, and D41.0-D41.1 for kidney cancer, C67-C67.9, D09.0, D30.3, D41.4-D41.8, D49.4, Z12.6-Z12.79, Z80.52, and Z85.51 for bladder cancer, and C61-C61.9, 185-185.9, V10.46, V16.42, and V76.44 for prostate cancer [6, 14].

Definition of smoking

These three urological cancers have a common and primary risk factor - smoking. Smoking as defined in GBD 2021 includes cigarettes, hand-rolled tobacco, cigars, and waterpipe (hookah), with the population stratified into current and former smokers based on self-reported smoking status in the past 12 months. While GBD 2021 does not directly include pack-years data, prior studies have shown that smoking intensity is significantly associated with increased risk of urological cancers (relative risk (RR) for bladder cancer: 1.52 (95% uncertainty interval (UI): 1.41-1.64) for ≥20 pack-years vs. non-smokers), supporting the biological plausibility of smoking-attributable burden estimates [9].

Statistical analysis

The raw count and ASRs were used for analysis in this study. The ASRs of YLDs, YLLs, and DALYs. ASRs (per 100,000 persons) were calculated by weighting age-specific rates with the GBD world population standard (2000 WHO standard) to eliminate distortions from age distribution differences, whereas raw counts represent the absolute number of cases within each stratum [15]. The 95%UI in GBD 2021 was calculated using Bayesian hierarchical models to address specification bias and data sparsity, particularly in low-resource regions. The change points of the temporal trend of the urological cancers were estimated by the Monte Carlo permutation test (1,000 iterations, p<0.05) using the Joinpoint Regression Program, Version 4.9.0.0 (developed by the National Cancer Institute and available as a free download). Change points were identified as inflection points where the annual percent change (APC) showed a statistically significant shift, with model selection based on the Akaike information criterion (AIC) and maximum segments set to 5. The APC was estimated for each temporal segment, and the average annual percent change (AAPC) was calculated as the geometric weighted average of APCs to characterize the overall trend from 1991 to 2021.

To address potential confounding variables, the analysis stratified data by SDI (composed of per capita GDP, education index, and life expectancy at birth), age, and gender, aligning with GBD's standard approach to control for socioeconomic and demographic variables. The SDI strata were defined as: high (SDI>0.85), high-middle (SDI=0.65-0.85), middle (SDI=0.45-0.65), low-middle (SDI=0.25-0.45), and low (SDI<0.25), consistent with prior GBD studies on urological cancers. The GBD methodology handles missing data through advanced statistical modeling, including Bayesian approaches, to impute values in regions with limited data, ensuring robust estimates even in low-resource settings.

## Results

The global burden of urological cancers attributed to smoking in 2021

The cases of kidney, bladder, and prostate cancers attributed to smoking were 0.16×10^5^ (95%UI: 0.09-0.25), 0.84×10^5^ (95%UI: 0.61-1.16), and 0.34×10^5^ (95%UI: 0.15-0.58) for YLDs in 2021, respectively (Tables 1-3). Among the different SDI regions, the high SDI region accounted for the greatest number of YLDs for urological cancers: 0.08×10^5^ (95%UI: 0.04-0.13), 0.34×10^5^ (95%UI: 0.24-0.46) and 0.19×10^5^ (95%UI: 0.08-0.33) for kidney, bladder, and prostate cancers, respectively, followed by high-middle and middle SDI regions. Furthermore, the cases of urological cancers were 3.67×10^5^ (95%UI: 2.24-5.13), 11.54×10^5^ (95%UI:9.73-13.76), and 2.48×10^5^ (95%UI:1.15-4.02) for YLLs in 2021, respectively, for kidney, bladder and prostate cancers. The cases of the urological cancers were 3.82×10^5^ (95%UI:2.34-5.37), 12.38×10^5^ (95%UI:10.44-14.78), and 2.82×10^5^ (95%UI:1.31-4.58) for DALYs in 2021, respectively, for kidney, bladder, and prostate cancers. Similar to YLDs, the high SDI region had the greatest number of YLLs and DALYs followed by high-middle and middle SDI regions (Table 1-3).

**Table 1:** The number and age-standardized rates (ASRs) of years lived with disability (YLDs), years of life lost (YLLs), and disability-adjusted life years (DALYs) for kidney cancer attributed to smoking in 2021 and the change from 1991 to 2021 among different SDI regions, age and gender. SDI: socio-demographic index; AAPC: average annual percent change; 95UI: 95% uncertainty interval.

	Kidney cancer								
	YLDs			YLLs			DALYs		
Location	Number (95UI:lower,upper) (100,000s) in 2021	ASRs (95UI:lower,upper) per 100,000 in 2021	AAPC (1991 to 2021)	Number (95UI:lower,upper) (100,000s) in 2021	ASRs (95UI:lower,upper) per 100,000 in 2021	AAPC (1991 to 2021)	Number (95UI:lower,upper) (100,000s) in 2021	ASRs (95UI:lower,upper) per 100,000 in 2021	AAPC (1991 to 2021)
Global	0.16(0.09,0.25)	0.18(0.11,0.29)	-0.17 (-0.32,-0.02)	3.67(2.24,5.13)	4.19(2.55,5.87)	-1.2(-1.33,-1.07)	3.82(2.34,5.37)	4.37(2.66,6.14)	-1.16 (-1.29,-1.04)
High SDI	0.08(0.04,0.13)	0.41(0.23,0.66)	-0.49 (-0.63,-0.34)	1.45(0.84,2.14)	7.28(4.28,10.62)	-1.71(-1.83,-1.6)	1.53(0.89,2.26)	7.68(4.54,11.29)	-1.66 (-1.78,-1.54)
High-middle SDI	0.05(0.03,0.09)	0.27(0.16,0.42)	0.94(0.69,1.19)	1.33(0.83,1.85)	6.61(4.14,9.2)	-0.48 (-0.66,-0.3)	1.38(0.87,1.91)	6.88(4.32,9.5)	-0.43 (-0.61,-0.25)
Middle SDI	0.02(0.01,0.03)	0.08(0.05,0.12)	1.87(1.69,2.06)	0.68(0.42,0.94)	2.43(1.5,3.37)	0.13(-0.12,0.38)	0.7(0.43,0.97)	2.51(1.55,3.48)	0.17(-0.08,0.42)
Low-middle SDI	0(0.01,0)	0.03(0.01,0.04)	1.03(0.86,1.2)	0.18(0.11,0.25)	1.22(0.76,1.74)	0.22(0.1,0.35)	0.18(0.11,0.26)	1.25(0.77,1.77)	0.24(0.11,0.37)
Low SDI	0(0,0)	0.01(0,0.01)	0.44(0.31,0.57)	0.02(0.01,0.04)	0.49(0.27,0.74)	-0.17 (-0.27,-0.07)	0.03(0.01,0.04)	0.5(0.28,0.75)	-0.16 (-0.26,-0.06)
Sex									
Male	0.14(0.08,0.21)	0.33(0.19,0.51)	0.01(-0.12,0.14)	3.25(1.99,4.52)	7.88(4.81,11.04)	-1.08 (-1.2,-0.96)	3.38(2.08,4.71)	8.21(5.01,11.5)	-1.04 (-1.16,-0.93)
Female	0.02(0.01,0.04)	0.05(0.03,0.08)	-1.11 (-1.26,-0.96)	0.42(0.25,0.62)	0.91(0.54,1.34)	-2.17 (-2.27,-2.06)	0.45(0.26,0.66)	0.96(0.57,1.43)	-2.12 (-2.22,-2.02)
Age									
30-54 years	0.03(0.02,0.04）	0.07(0.04,0.12)	-0.12 (-0.26,0.02)	0.58(0.37,0.79)	1.54(0.97,2.09)	-1.15 (-1.35,-0.94)	0.61(0.38,0.82)	1.61(1.02,2.18)	-1.1 (-1.31,-0.91)
55+ years	0.13（0.08,0.21)	0.89(0.51,1.4)	0(-0.1,0.09)	3.09(1.87,4.36)	20.79(12.6,29.35)	-1.06 (-1.17,-0.94)	3.22(1.95,4.55)	21.68 (13.15,30.63)	-1.02 (-1.13,-0.91)

**Table 2:** The number and age-standardized rates (ASRs) of years lived with disability (YLDs), years of life lost (YLLs) and disability-adjusted life years (DALYs) for bladder cancer attributed to smoking in 2021 and the change from 1991 to 2021 among different SDI regions, age and gender. SDI: socio-demographic index; AAPC: average annual percent change; 95UI: 95% uncertainty interval.

	Bladder cancer								
	YLDs			YLLs			DALYs		
Location	Number (95UI:lower,upper) (100,000s) in 2021	ASRs (95UI:lower,upper) per 100,000 in 2021	AAPC (1991 to 2021)	Number (95UI:lower,upper) (100,000s) in 2021	ASRs (95UI:lower,upper) per 100,000 in 2021	AAPC (1991 to 2021)	Number (95UI:lower,upper) (100,000s) in 2021	ASRs (95UI:lower,upper) per 100,000 in 2021	AAPC (1991 to 2021)
Global	0.84(0.61,1.16)	0.97(0.7,1.33)	-0.69 (-0.82,-0.55)	11.54(9.73,13.76)	13.36 (11.26,15.98)	-1.79 (-1.93,-1.65)	12.38 (10.44,14.78)	14.33 (12.09,17.14)	-1.72 (-1.86,-1.59)
High SDI	0.34(0.24,0.46)	1.65(1.16,2.25)	-0.89 (-1.07,-0.71)	3.41(2.85,4)	16.16(13.66,18.8)	-2.01 (-2.23,-1.79)	3.75(3.13,4.40)	17.81 (15.02,20.78)	-1.92 (-2.14,-1.7)
High-middle SDI	0.3(0.33,0.41)	1.48(1.04,2.05)	-0.21 (-0.33,-0.09)	3.93(3.32,4.7)	19.59(16.5,23.42)	-1.66 (-1.92,-1.41)	4.23(3.56,5.06)	21.06 (17.71,25.17)	-1.57 (-1.89,-1.26)
Middle SDI	0.16(0.11,0.23)	0.57(0.39,0.83)	0.66(0.55,0.78)	2.8(2.21,3.64)	10.55(8.35,13.69)	-1.34 (-1.53,-1.15)	2.95(2.34,3.85)	11.12(8.83,14.45)	-1.27 (-1.45,-1.09)
Low-middle SDI	0.04(0.03,0.07)	0.3(0.2,0.47)	-0.18 (-0.32,-0.05)	1.12(0.91,1.59)	8.02(6.49,11.42)	-1.22 (-1.34,-1.09)	1.17(0.94,1.66)	8.33(6.73,11.87)	-1.19 (-1.31,-1.06)
Low SDI	0.01(0.01,0.01)	0.15(0.1,0.21)	-0.17 (-0.32,-0.03)	0.26(0.21,0.32)	5.38(4.36,6.78)	-0.87 (-1.04,-0.7)	0.26(0.21,0.33)	5.53(4.47,6.94)	-0.86 (-1,0-0.71)
Sex									
Male	0.76(0.54,1.04)	1.88(1.35,2.57)	-0.7 (-0.75,-0.65)	10.5(8.82,12.53)	26.65(22.35,31.96)	-1.83 (-1.93,-1.72)	11.26(9.45,13.48)	28.52(23.9,34.2)	-1.74 (-1.87,-1.63)
Female	0.08(0.06,0.12)	0.18(0.13,0.25)	-1.35 (-1.45,-1.24)	1.03(0.86,1.23)	2.23(1.85,2.65)	-2.2(-2.25,-2.15)	1.12(0.92,1.33)	2.41(1.99,2.85)	-2.17 (-2.25,-2.1)
Age									
30-54 years	0.1(0.07,0.14)	0.26(0.18,0.37)	-0.3(-0.42,-0.18)	1.22(1.04,1.45)	3.24(2.75,3.84)	-1.68 (-1.82,-1.54)	1.32(1.12,1.56)	3.5(2.96,4.15)	-1.59 (-1.73,-1.45)
55+ years	0.75(0.54,1.02)	5.02(3.6,6.84)	-0.59 (-0.65,-0.54)	10.32(8.7,12.36)	69.45 (58.58,83.21)	-1.65 (-1.77,-1.52)	11.07(9.37,13.26)	74.46 (63.04,89.22)	-1.59 (-1.67,-1.5)

**Table 3:** The number and age-standardized rates (ASRs) of years lived with disability (YLDs), years of life lost (YLLs) and disability-adjusted life years (DALYs) for prostate cancer attributed to smoking in 2021 and the change from 1991 to 2021 among different SDI regions, age and gender. SDI: socio-demographic index; AAPC: average annual percent change; 95UI: 95% uncertainty interval.

	Prostate cancer								
	YLDs			YLLs			DALYs		
Location	Number (95UI:lower,upper) (100,000s) in 2021	ASRs (95UI:lower,upper) per 100,000 in 2021	AAPC (1991 to 2021)	Number (95UI:lower,upper) (100,000s) in 2021	ASRs (95UI:lower,upper) per 100,000 in 2021	AAPC (1991 to 2021)	Number (95UI:lower,upper) (100,000s) in 2021	ASRs (95UI:lower,upper) per 100,000 in 2021	AAPC (1991 to 2021)
Global	0.34(0.15,0.58)	0.39(0.17,0.67)	-0.91 (-1.03,-0.78)	2.48(1.15,4.02)	2.87(1.33,4.67)	-1.96 (-2.04,-1.88)	2.82(1.31,4.58)	3.26(1.52,5.33)	-1.84 (-1.95,-1.74)
High SDI	0.19(0.08,0.33)	0.93(0.4,1.62)	-1.14 (-1.31,-0.97)	0.8(0.37,1.33)	3.7(1.72,6.05)	-2.9 (-3.06,-2.74)	0.99(0.45,1.63)	4.63(2.14,7.56)	-2.62 (-2.78,-2.46)
High-middle SDI	0.09(0.04,0.15)	0.43(0.19,0.72)	0.65(0.48,0.82)	0.69(0.32,1.09)	3.42(1.58,5.41)	-1.27 (-1.46,-1.09)	0.78(0.36,1.23)	3.86(1.77,6.05)	-1.12 (-1.25,-0.99)
Middle SDI	0.05(0.02,0.08)	0.17(0.07,0.29)	1.17(1.03,1.31)	0.6(0.27,0.94)	2.26(1.03,3.61)	-0.93 (-1.08,-0.78)	0.64(0.3,1.01)	2.43(1.11,3.89)	-0.82 (-0.96,-0.67)
Low-middle SDI	0.02(0,0.03)	0.11(0.05,0.19)	1.43(1.23,1.63)	0.3(0.14,0.5)	2.22(0.99,3.54)	-0.21 (-0.39,-0.03)	0.31(0.14,0.5)	2.33(1.04,3.74)	-0.16 (-0.23,-0.1)
Low SDI	0(0,0)	0.05(0.02,0.09)	0.51(0.43,0.6)	0.08(0.03,0.14)	1.76(0.74,2.91)	-1.96 (-2.04,-1.88)	0.09(0.04,0.14)	1.82(0.76,2.99)	-1.84 (-1.95,-1.74))
Sex									
Male	0.34(0.15,0.58)	0.39(0.17,0.67)	-0.91 (-1.03,-0.78)	2.48(1.15,4.02)	2.87(1.33,4.67)	-0.45(-0.49,-0.4)	2.82(1.31,4.58)	3.26(1.52,5.33)	-0.43 (-0.47,-0.39)
Age									
30-54 years	0.02(0.01,0.04)	0.06(0.02,0,.1)	0.62(0.44,0.8)	0.16(0.07,0.24)	0.42(0.18,0.65)	-1.15 (-1.31,-0.99)	0.18(0.08,0.28)	0.48(0.21,0.73)	-0.97 (-1.1,-0.84)
55+ years	0.32(0.14,0.55)	2.16(0.93,3.68)	-0.86 (-0.95,-0.77)	2.32(1.07,3.779)	15.61(7.23,25.49)	-1.86 (-1.92,-1.8)	2.64(1.23,4.33)	17.77(8.25,29.12)	-1.75 (-1.82,-1.69)

Moreover, the ASRs of YLDs for the kidney, bladder, and prostate cancers attributed to smoking were 0.18 (95%UI:0.17-0.67), 0.97 (95%UI:0.7-1.33), and 0.39 (95%UI:0.17-0.58) in 2021, respectively. The ASRs of YLLs and DALYs for these urological cancers were 4.19 (95%UI:2.55-5.87) and 4.37 (95%UI:2.66-6.14) (kidney cancer), 13.36 (95%UI:11.26-15.98) and 14.33 (95%UI:12.09-17.14) (bladder cancer), and 2.87 (95%UI:1.33-4.67) and 3.26 (95%UI:1.52-5.33) (prostate cancer) in 2021, respectively. Among the different SDI regions, the high-SDI region also had the highest ASRs of YLDs, YLLs, and DALYs for these three urological cancers, followed by high-middle and middle SDI regions (Table 1-3).

Moreover, there were significant differences in the YLDs, YLLs, and DALYs of the urological cancers in terms of gender and age. The number and ASRs of YLDs, YLLs, and DALYs for kidney cancer in men attributed to smoking were almost seven to eight times higher than that in women (Table 1). The number and ASRs of YLDs, YLLs, and DALYs for bladder cancer in men were almost nine to 10 times that of women (Table 2). Furthermore, the number of YLDs, YLLs, and DALYs for kidney cancer attributed to smoking among people aged 55 years was four to six times higher than for those below 54 years, while the ASRs among those aged 55 years were almost 12 times higher (Table 1). The number of bladder cancer cases among those aged 55 years was seven to nine times higher than for those below 54 years, while the ASRs were 19 to 21 times higher (Table 2). Finally, the number of prostate cancer cases among those aged 55 years was 14 to 16 times higher, while the ASRs were 36 to 37 times higher than for those aged 54 years and younger (Table 3).

The global trend of urological cancers attributed to smoking among different SDI regions from 1991 to 2021

For kidney cancer, the AAPC of ASRs of YLDs from 1991 to 2021 on a global scale (-0.17, 95% confidence interval (CI):-0.32- to -0.02) and in high-SDI region (-0.49, 95%CI:-0.63- to -0.34) showed a decreasing trend, while that in others displayed an increasing trend (0.94, 95%CI:0.69,1.19; 1,87, 95%CI:1.69-2.06; 1.03, 95%CI:0.86,1.2, and 0.44, 95%CI:0.31-0.57). Furthermore, only the AAPC of ASRs of YLLs and DALYs in low-middle SDI region (0.22, 95%CI:0.1-0.35 and 0.24, 95%CI:0.11-0.37) significantly increased from 1991 to 2021, while others except in the middle SDI region showed a significantly decreasing trend (Table 1). For bladder cancer, the AAPC of ASRs of YLDs, YLLs, and DALYs in all regions showed a significantly downward trend (Table 2). For prostate cancer, except the AAPC of ASRs of YLDs in high-middle (0.65, 95%CI:0.48-0.82), middle (1.17, 95CI:1.03-1.31), low-middle (1.43, 95%CI:1.23-1.63), and low-SDI regions (0.51,95%CI:0.17-0.67) was steadily increasing, while that in others showed a downward trend (Table 3). In addition, the change of YLDs, YLLs, and DALYs for urological cancers in high-SDI region had the greatest decline among all regions.

**Figure 1:**
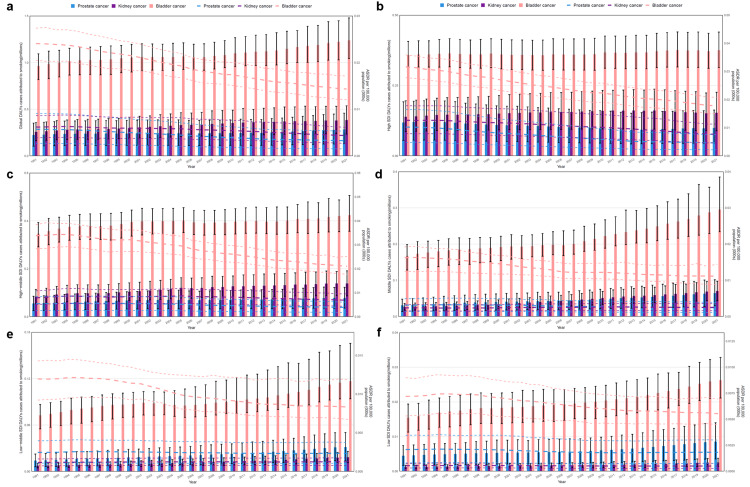
The temporal trend of disability-adjusted life years (DALYs) for the urological cancers attributed to smoking among different SDI regions from 1991 to 2021. (a)-(f) The temporal trend of DALYs for urological cancers attributed to smoking from 1991 to 2021 on a global scale in high, high-middle, middle, low-middle, and low SDI regions. The bar chart shows that the number of DALYs for urological cancers (millions). The dashed line shows the ASRs of DALYs for urological cancers per 100,000 people. Image credit: Zewei Liu.

The number and ASRs of DALYs for bladder cancer attributed to smoking were the highest in the world and among the different SDI levels (Figure 1a). The ASRs and number of DALYs for kidney cancer were higher than that of prostate cancer in high and high-middle SDI regions, and middle SDI region after 2007 (Figure 1b-d), while the ASRs and number showed an opposite trend in low and low-middle SDI regions and middle SDI region before 2007 (Figure 1d-f).

On a global scale, the number of DALYs for bladder cancer attributed to smoking from 1991 to 2021 showed the most remarkable increasing trend, followed by kidney cancer, while an obvious small increase in that of prostate cancer can be noted (Figure 1a). In the high-SDI region, the number of urological cancers showed no obvious variation in trends (Figure 1b). In the high-middle SDI region, there was a significant increasing trend from 1991 to 1995, and after that, there was no obvious change (Figure 1c). In the middle SDI region, there was a small change before 2007, but a significant increase was observed after 2007 (Figure 1d). Furthermore, in low-middle and low SDI regions, the number of bladder cancer and prostate cancer cases increased significantly from 1991 to 2021, while the number of kidney cancer cases showed insignificant changes in the trend (Figure 1e,f).

Despite the increased number of DALYs for urological cancers attributed to smoking across the world, the ASRs showed a decreasing trend for urological cancers, especially bladder cancer (Figure 1a). The trends for urological cancers showed a more obvious decline in regions with a higher SDI, while there was no obvious downward trend in low-middle and low SDI regions (Figure 1b-f).

The temporal trend of urological cancers attributed to smoking stratified by gender

**Figure 2:**
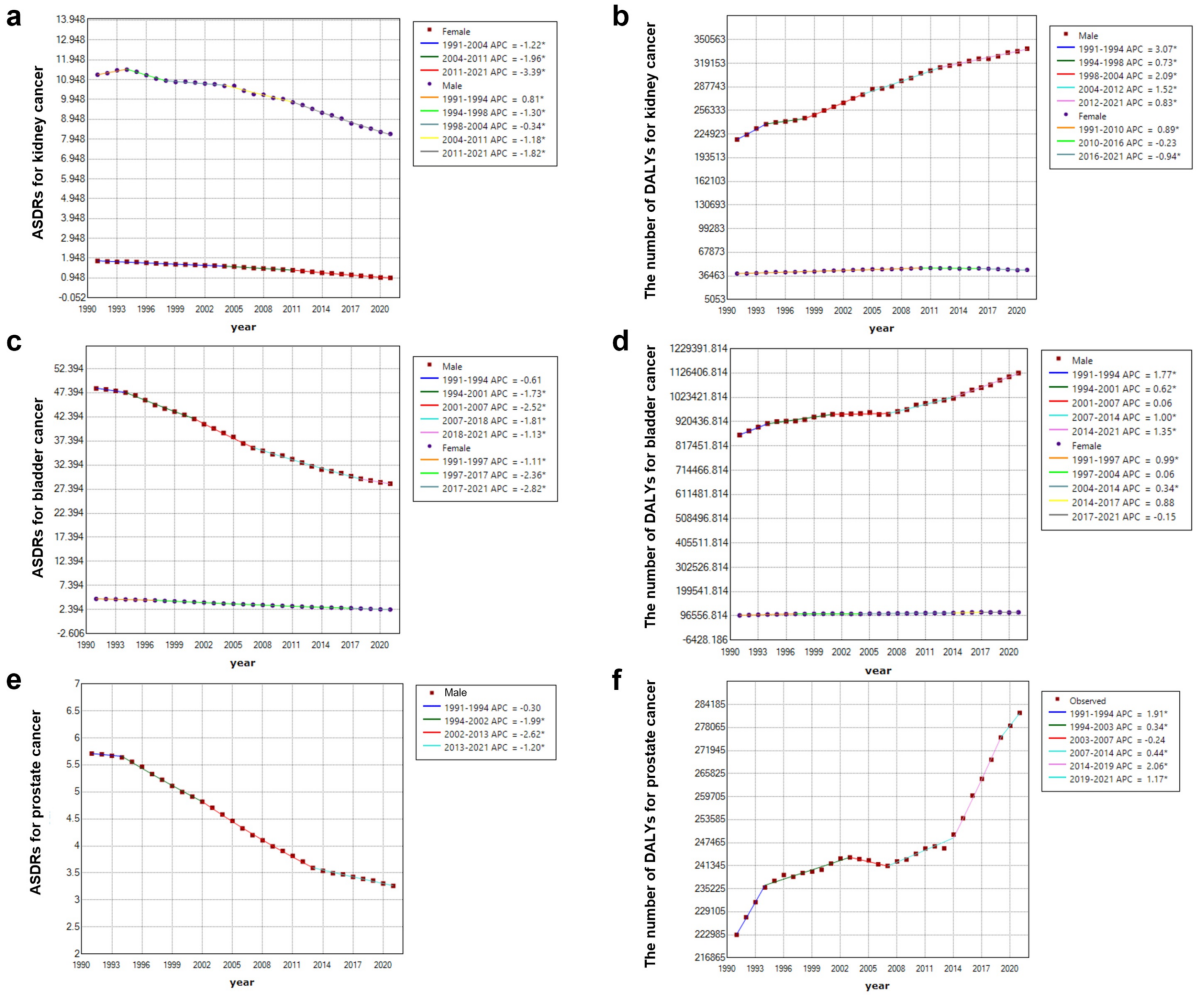
The temporal trend of age-standardized rates (ASRs) and number of disability-adjusted life years (DALYs) for urological cancers attributed to smoking stratified by gender on a global scale. (a,b) The ASRs and number of DALYs attributed to smoking stratified by gender on a global scale from 1991 to 2021 for kidney cancer. (c,d) The ASRs and number of DALYs attributed to smoking stratified by gender on a global scale from 1991 to 2021 for bladder cancer. (e,f) The ASRs and number of DALYs attributed to smoking stratified by gender on a global scale from 1991 to 2021 for prostate cancer. The ASRs are the rates per 100,000 people. Image credit: Zewei Liu.

For kidney and bladder cancers, the AAPC of ASRs attributed to smoking generally decreased in men and women. Moreover, the degree of decline in women was much greater than that in men, while the degree of decline in men for kidney cancer was the smallest (Tables 1,2). To figure out the precise temporal trend of DALYs for urological cancers, as shown in Figure 2, we measured the change points using Joinpoint. For kidney cancer, the temporal trend of ASDRs in women continuously decreased from 1991 to 2021 with the greatest decline from 2011 to 2021 (-3.39, 95%CI: -3.55 to -3.23).

The APC of ASDRs in men from 1991 to 1994 showed an increasing trend (APC: 0.81, 95%CI: 0.2-1.44), while from 1994 to 2021, it decreased in men, with the greatest decline from 2011 to 2021 (APC:-1.82, 95%CI: -1.92 to -1.73) as well (Figure 2a). Contrary to the ASDRs, the number of DALYs attributed to smoking increased from 1991 to 2021, while that in men (AAPC: 1.47, 95%CI: 1.36-1.59), the increase was larger than that in women (AAPC: 0.36, 95%CI: 0.24-0.47). The increase in the number of DALYs in men mainly occurred between 1991 and 1994 and between 1998 and 2012. For women, the increase in DALYs primarily took place from 1991 to 2010, and a downward trend began to emerge from 2010 to 2021 (Figure 2b).

For bladder cancer, the ASDRs also showed a downward trend in men and women from 1991 to 2021. The decline in male ASDRs was mainly concentrated from 1994 to 2018, and it became slightly milder after 2018. In contrast, the decline in women showed a trend of accelerating year on year (Figure 2c). In addition, the number of DALYs for both men (AAPC: 0.88, 95%CI: 0.79-0.98) and women (AAPC: 0.39, 95%CI: 0.25-0.53) showed an increasing trend from 1991 to 2021. For men, the main increases occurred from 1991 to 1994 and from 2014 to 2021. However, for women, there was a downward trend from 2017 to 2021 (Figure 2d).

Lastly, only men get prostate cancer. From 1991 to 2021, ASDRs for men showed a downward trend, with the main decline occurring between 2002 and 2013 (Figure 2e). In addition, the number of DALYs for males (AAPC: 0.78, 95%CI:0.69-0.87) generally showed an upward trend, with the main increases occurring from 1991 to 1994 and from 2014 to 2019 (Figure 2f).

The temporal trend of urological cancers attributed to smoking stratified by gender

As shown in Tables 1-3, the three types of urological cancers attributed to smoking showed no recorded cases before the age of 30 years. From 1991 to 2021, ASRs of YLLs and DALYs generally exhibited a downward trend. However, YLDs for prostate cancer (0.62, 95%CI: 0.44-0.8) in the 30-54 age group showed a significant upward trend.

**Figure 3:**
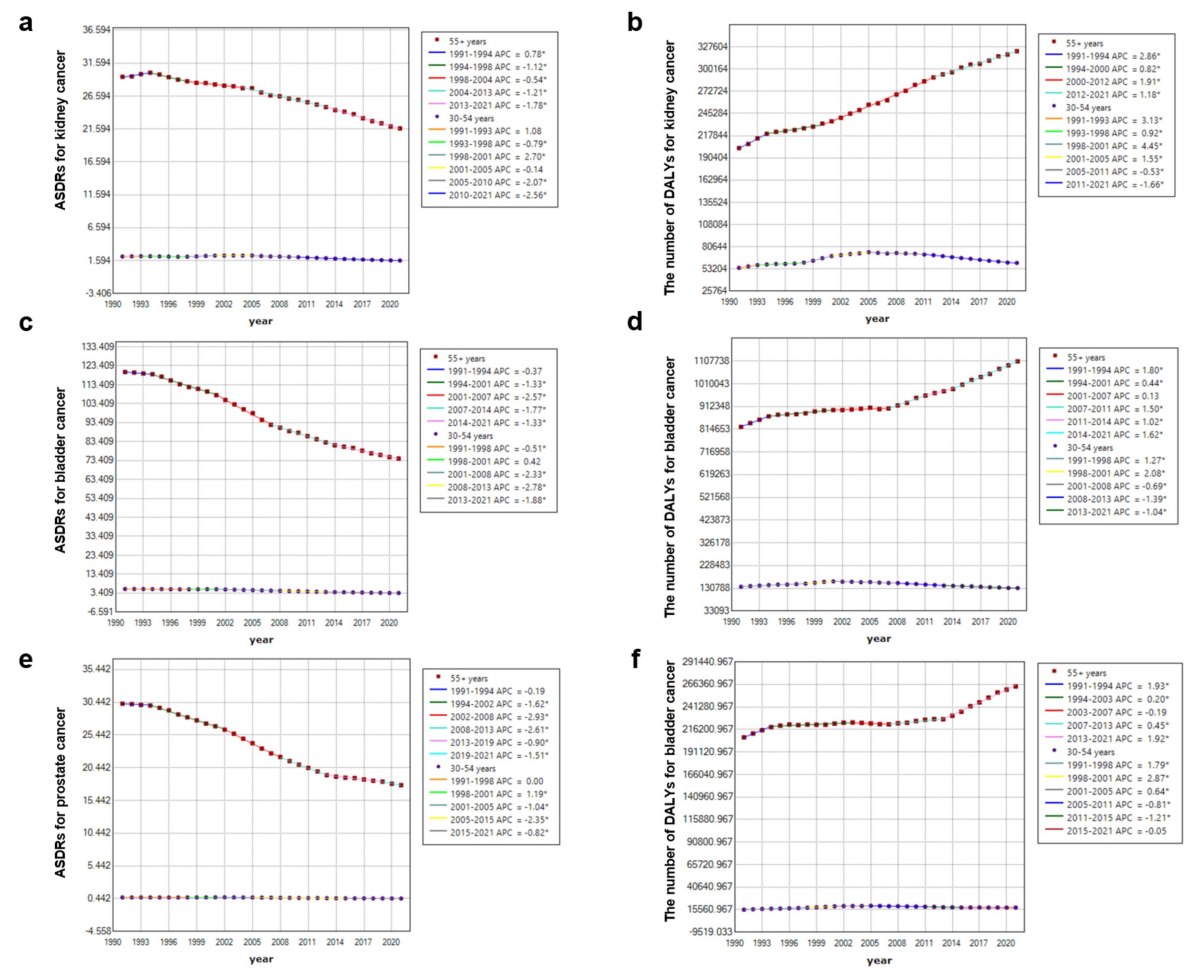
The temporal trend of age-standardized rates (ASRs) and number of disability-adjusted life years (DALYs) for urological cancers attributed to smoking stratified by age on a global scale. (a,b) The ASRs and number of DALYs attributed to smoking stratified by age on a global scale from 1991 to 2021 for kidney cancer. (c,d) The ASRs and number of DALYs attributed to smoking stratified by age on a global scale from 1991 to 2021 for bladder cancer. (e,f) The ASRs and number of DALYs attributed to smoking stratified by age on a global scale from 1991 to 2021 for prostate cancer. The ASRs are the rates per 100,000 people. Image credit: Zewei Liu.

Furthermore, as shown in Figure 3, for kidney cancer, ASDRs attributed to smoking generally showed a downward trend in both age groups (older than 55 years and those in the 30-54 age group) from 1991 to 2021 (AAPC: -1.1, 95%CI: -1.31- to -0.91; AAPC: -1.02, 95%CI: -1.13 to -0.91), and the downward trend gradually increased after 2004 (Figure 3a). From 1991 to 2021, the overall number of DALYs in both groups increased (AAPC: 0.33, 95%CI: 0.19-0.28; AAPC: 1.56, 95%CI: 1.48-1.65). Among them, the group aged 55 years or older showed a continuous upward trend, while the group aged 30-54 years presented a gradual downward trend after 2005 (Figure 3b).

For bladder cancer, the overall trend of ASDRs attributed to smoking in both age groups showed a downward trend, and the extent of decline was greater than that of kidney cancer (AAPC: -1.59, 95%CI: -1.73- to -1.45, -1.59, 95%CI: -1.67- to -1.5). The decline in ASDRs primarily occurred after 2001 (Figure 3c). In addition, from 1991 to 2021, the number of DALYs in the over-55 age group showed a significant upward trend (AAPC: 0.99, 95%CI: 0.86-1.11), while those in the 30-54 age group exhibited a slight overall decline (AAPC: -0.17, 95%CI: -0.33 to -0.02). The number of DALYs in the over-55 age group showed a dominant increasing trend from 1991 to 1994 and after 2007. In contrast, the DALYs in the 30-54 age group gradually declined after 2001 (Figure 3d).

Lastly, for prostate cancer, ASDRs attributed to smoking showed a downward trend in both age groups from 1991 to 2021, with the decline in the over-55 group (AAPC: -1.75, 95%CI: -1.81 to -1.69) being significantly greater than that in the 30-54 group (AAPC: -0.97, 95%CI: -1.1 to -0.84). Moreover, the decline in ASDRs for the 30-54 age group began after 2001 (Figure 3e). In addition, from 1991 to 2021, the number of DALYs attributed to smoking showed an increasing trend in both age groups, with an upward trend in the over-55 group (AAPC: 0.83, 95%CI: 0.74-0.92) being significantly more pronounced than that in the 30-54 group (AAPC: 0.45, 95%CI: 0.28-0.62). For the over-55 group, the growth was primarily concentrated in two periods: 1991-1994 and 2013-2021. For the 30-54 age group, the increasing trend gradually decelerated over the years and turned into a downward trend after 2005 (Figure 3f).

The global burden of urological cancers attributed to smoking among SDI regions in 2021

**Figure 4:**
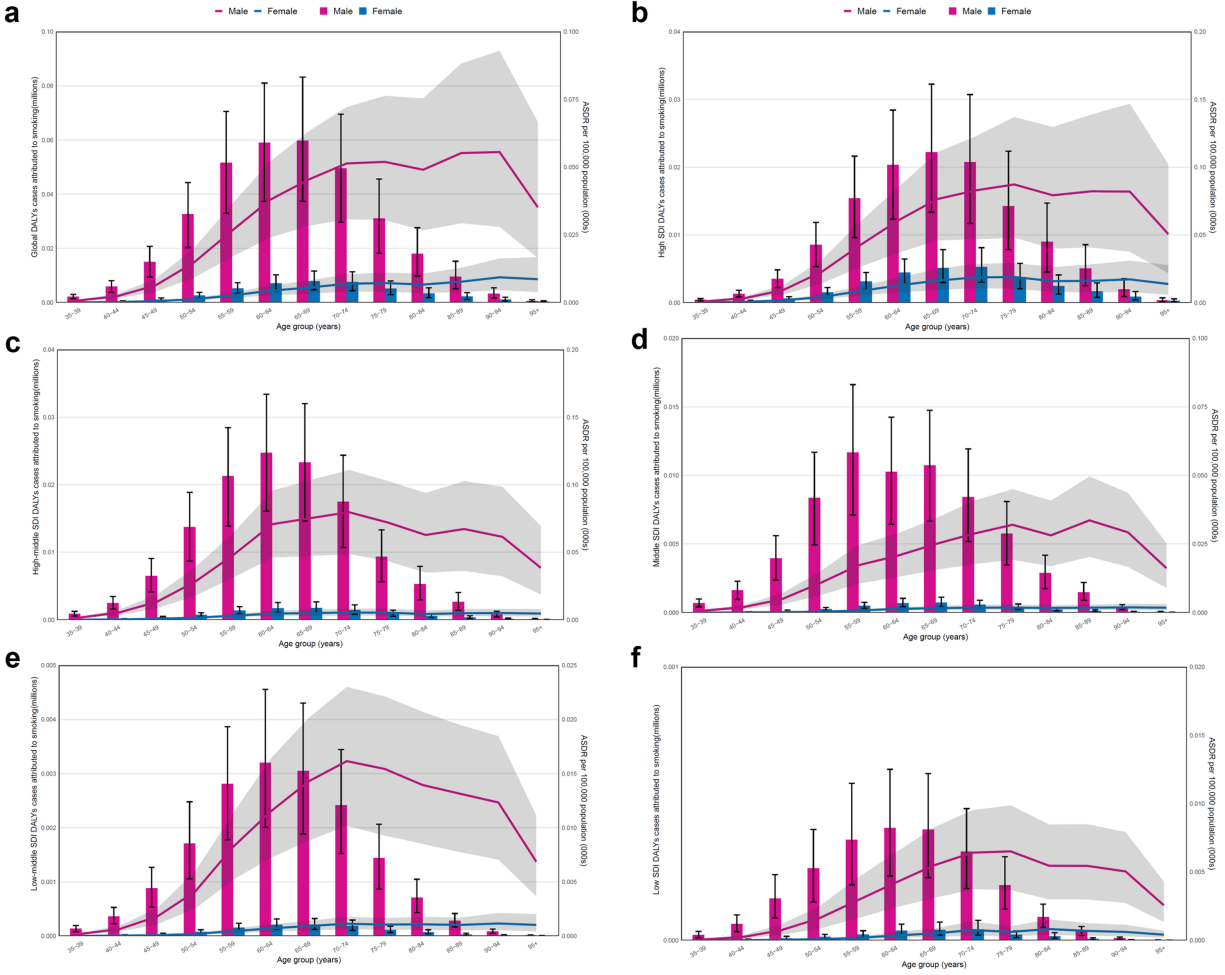
The age-standardized rates (ASRs) and number of disability-adjusted life years (DALYs) for kidney cancer attributed to smoking stratified by age and gender in 2021. (a)-(f) The burden of DALYs for kidney cancer attributed to smoking stratified by age and genders on a global scale, high, high-middle, middle, low-middle and low socio-demographic index (SDI), respectively. The bar chart shows the number of DALYs for kidney cancer (millions). The line shows the ASRs of DALYs for kidney cancer per 100,000 people. Image credit: Zewei Liu.

For kidney cancer, the number of DALYs attributed to smoking occurred after 35 years of age and was primarily concentrated in the 65-69 age group for both men and women, while ASDRs were highest in the 90-95 age group worldwide (Figure 4a). Among the SDI regions, the male-to-female ratio of DALYs decreased with the increase of SDI. In high-SDI regions, the peak number of DALYs was concentrated in the 65-69 age group. In the middle SDI regions, the peak mainly occurred in the 55-59 and 65-69 age groups. In the remaining regions, the peak was concentrated in the 60-64 age group. Furthermore, ASDRs attributed to smoking were generally concentrated in the 70-79 age group in different SDI regions (Figure 4b-f).

**Figure 5:**
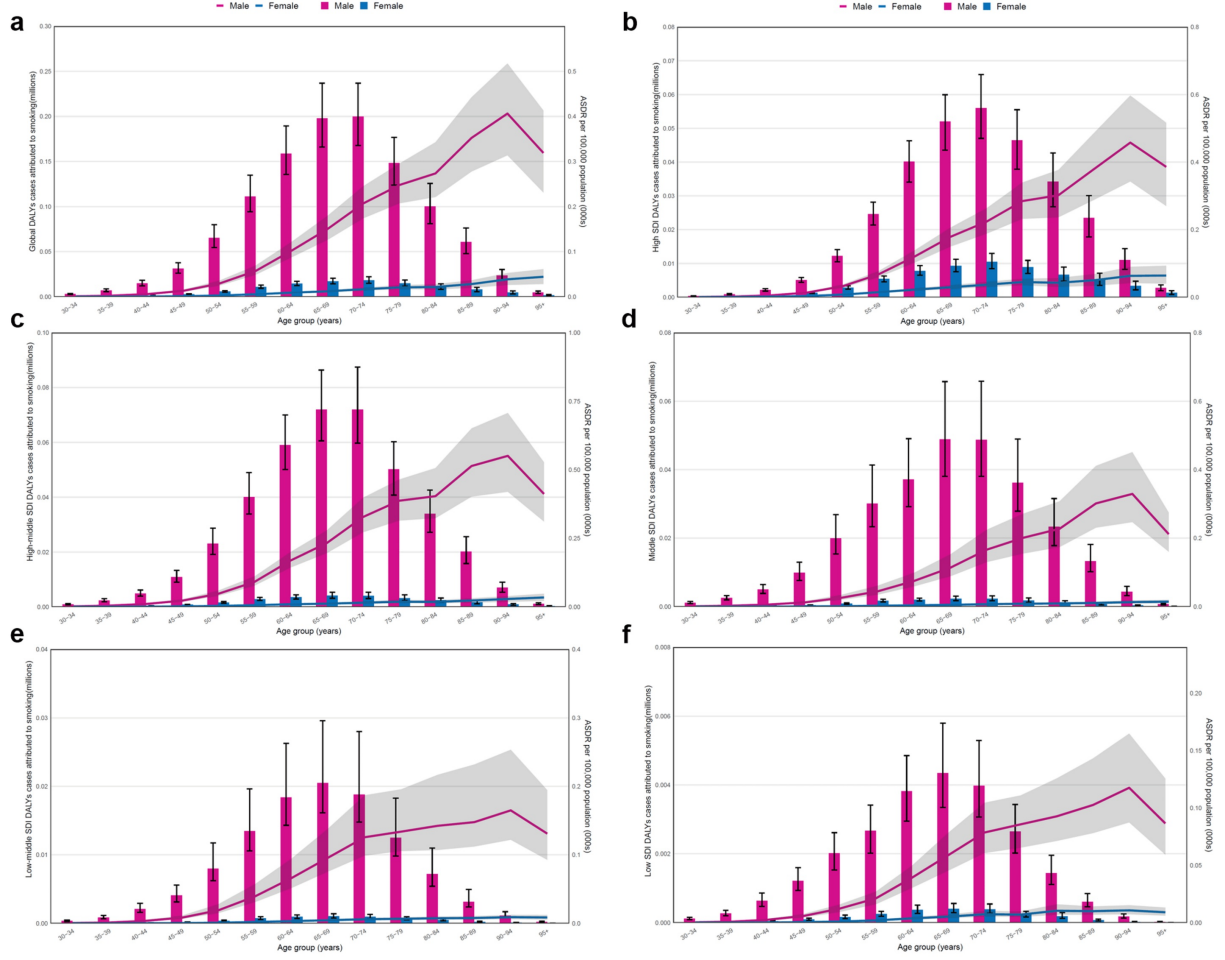
The age-standardized rates (ASRs) and number of disability-adjusted life years (DALYs) for bladder cancer attributed to smoking stratified by age and gender in 2021. (a)-(f) The burden of DALYs for bladder cancer attributed to smoking stratified by age and gender on a global scale and in high, high-middle, middle, low-middle and low SDI, respectively. The bar chart shows the number of DALYs for bladder cancer (millions). The line shows the ASRs of DALYs for bladder cancer per 100,000 people. Image credit: Zewei Liu.

For bladder cancer, the ASDRs attributed to smoking occurred after 30 years of age and showed an increasing trend with age, while the number of DALYs was primarily concentrated in the 65-74 age group worldwide (Figure 5a). Moreover, the male-to-female ratio of DALYs decreased, and the peak number of DALYs was concentrated in the 70-74 age group with the increase of SDI, with others in the 65-69 age group. In regions with a high SDI, ASDRs showed an increasing trend with age. However, in regions with a low SDI, this trend was relatively gentle. Furthermore, the ASDRs of women showed a trend of increasing with age, while the ASDRs of men showed a straight-line decrease above the age of 95 years (Figure 5b-f).

**Figure 6:**
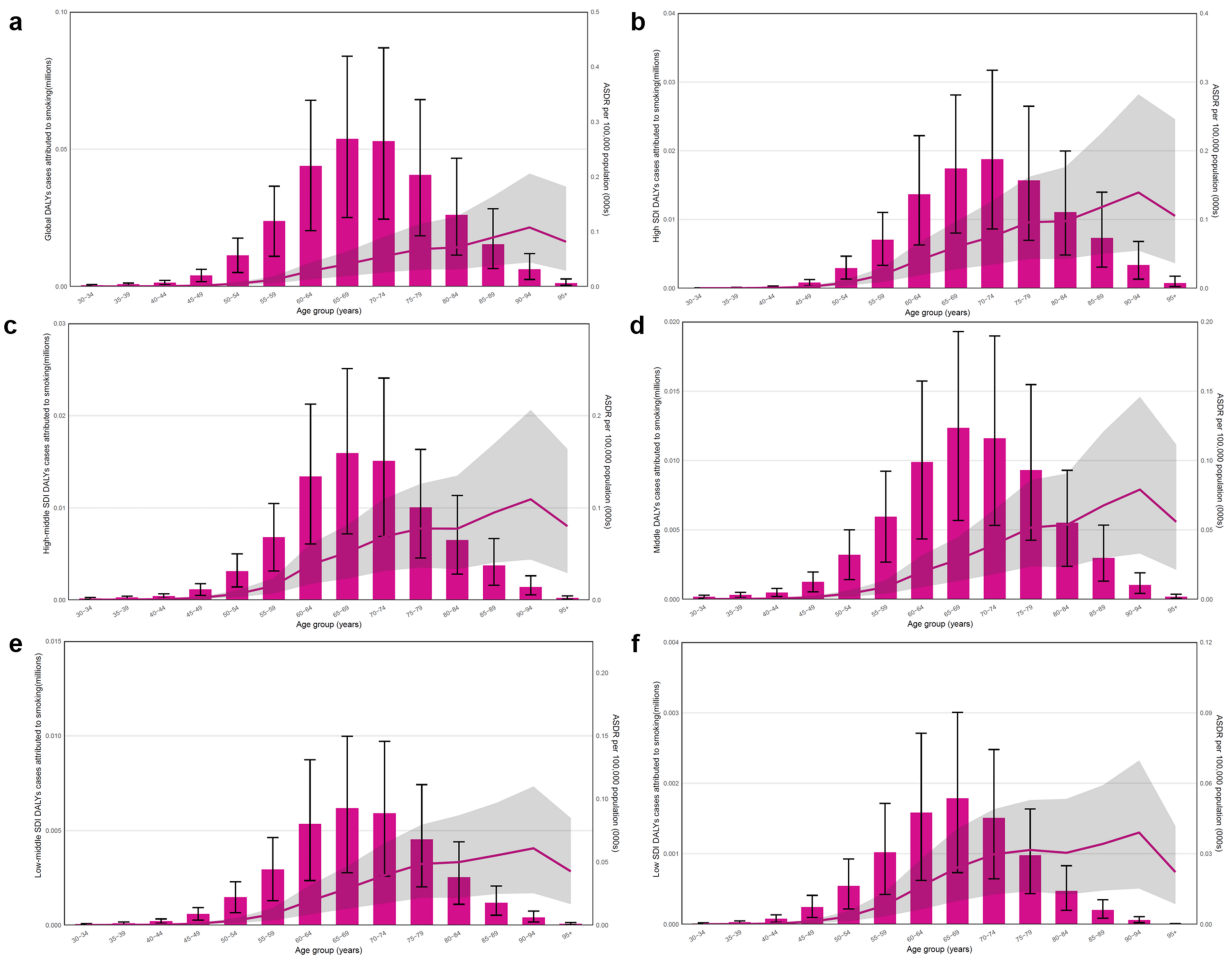
The age-standardized rates (ASRs) and number of disability-adjusted life years (DALYs) for prostate cancer attributed to smoking stratified by age in 2021. (a)-(f) The burden of DALYs for prostate cancer attributed to smoking stratified by age on a global scale in high, high-middle, middle, low-middle and low SDI, respectively. The bar chart shows the number of DALYs for prostate cancer (millions). The line shows the ASRs of DALYs for prostate cancer per 100,000 people. Image credit: Zewei Liu.

Lastly, for prostate cancer, the ASRs and number of DALYs attributed to smoking also occurred after 30 years of age and showed a similar trend with bladder cancer worldwide (Figure 6a). Moreover, the peak number of DALYs was also concentrated in the 70-74 age group with the increase of SDI, but in others, it was high in the 65-69 age group. In regions with a high SDI, ASDRs showed an increasing trend with age. However, in regions with a low SDI, this trend was relatively gentle (Figure 6b-f).

## Discussion

The present study comprehensively analyzed the temporal trend of YLDs, YLLs, and DALYs for kidney, bladder, and prostate cancers attributed to smoking on a global scale stratified by age, gender, and different SDI regions. The findings indicated that despite the decreased ASRs of YLLs and DALYs, the number of DALYs attributed to smoking still showed an increasing trend across the world. In addition, the epidemiological characteristics of urological cancers showed significant disparities among gender, age, and SDI levels. Our current findings will provide a theoretical foundation and supporting data for countries and regions worldwide to prevent urological cancers, especially those attributed to smoking.

Our findings showed that in kidney and prostate cancer, the burden of DALYs was most severe in the high-SDI region, followed by the high-middle SDI region. However, for bladder cancer, the heaviest burden was observed in the high-middle SDI region, followed by the high-SDI region, which was consistent with previous studies [3, 10, 14, 16, 17]. Notably, the study observed a paradox where ASRs declined while absolute DALYs increased in high-SDI regions. The contradiction likely stems from multiple interacting factors. While population aging and treatment advancements including targeted therapies for cancers extend patient survival and lower age-standardized mortality, the absolute burden may rise due to growing population size and longer disease durations. Additionally, improved cancer registration and diagnostic accuracy in high-SDI areas could inflate absolute case counts by capturing previously undiagnosed cases, even as preventive measures reduce incidence rates.

Moreover, ASRs of DALYs in the low-SDI region were the lowest in the world, which was contrary to conventional wisdom. The most likely possibilities were that the diagnosis of urological cancers in low and low-middle regions was not clear, as people in these regions were not active in seeking treatment for the disease in hospitals, and the statistical surveys on the disease were not detailed and accurate [18, 19]. On the other hand, the overall trend of ASDRs for urological cancers attributed to smoking showed a downward trend, especially with the increase of SDI. The decline became more obvious, consistent with previous studies [9, 20]. This indicated that regions with higher SDI had strengthened the prevention of urological cancers, particularly smoking cessation education.

However, the number of DALYs for urological cancers attributed to smoking basically showed an upward trend from 1991 to 2021. These observations, on the one hand, indicated that the number of urological cancer patients continued to rise, meaning global healthcare professionals and policymakers must step up their efforts for disease prevention. On the other hand, these also suggested that ongoing innovations in treatment methods were extending the lifespan of patients [21-24]. This also fully demonstrated that although the susceptibility to urological cancers attributed to smoking among those aged over 55 was higher than that of the 30-54 age group, the sustained and significant increase in DALYs for urological cancers also reflected ongoing innovations in disease treatment methods. In addition, consistent with the previous study, the ASRs and the number of DALYs for urological cancers attributed to smoking showed a trend of younger onset as the SDI decreased, with an approximate age difference of five years. This indicated that regions with relatively higher SDI have carried out more in-depth education on urological cancer prevention, especially regarding smoking issues [9, 25].

The ASRs and DALYs for urological cancers attributed to smoking showed significant gender disparities, driven by social, biological, and healthcare factors. Worldwide, men have higher smoking rates due to historical gender norms, with low SDI regions showing a 65% men vs. 12% women smoking prevalence [9]. Biologically, androgen pathways in males may enhance cancer susceptibility, while estrogen may offer females some protection. In low-SDI areas, smoking as a male social behavior and limited female screening worsen outcomes, whereas high-SDI regions have reduced gender smoking ratios (38% decline since 2000) and more balanced screening. These factors combine to amplify the male urological cancer burden across the SDI strata [26, 27].

Some limitations still remain in our current study, which were consistent with the GBD 2021 platform. Firstly, the methods used for data harvesting and analysis in GBD 2021 were different from the local epidemiological surveys. Specifically, the estimates from the GBD model for low-SDI regions may underestimate the true incidence of urological cancers due to underreporting as a result of incomplete medical records, which could lead to systematic biases in smoking-attributable burden calculations [3, 13]. Moreover, as previously discussed, there are still significant deficiencies in disease diagnosis and epidemiological investigations in regions with low SDI, where the lack of standardized cancer registries and diagnostic tools leads to misclassification of tumor types, potentially weakening the observed association between smoking and cancer risk. These factors may lead to biases in the conclusions drawn in these areas, particularly affecting the accuracy of temporal trend analyses and regional burden comparisons. Therefore, strengthening medical interventions and accurate statistical analysis of epidemiological data in these regions will ensure increased healthiness of people in these areas and will promote healthy development of the entire world in general.

## Conclusions

The global burden of YLDs, YLLs, and DALYs for kidney, bladder, and prostate cancers attributed to smoking still poses a threat to people across the world. The temporal and spatial trend of urological cancers showed significant disparities among age, gender, and different SDI regions. Our findings highlight the critical need to devise tailored prevention and intervention strategies. In high-SDI regions, efforts should focus on long-term smoking cessation maintenance programs and integrated cancer survivorship care to address the burden of chronic disability. In high-middle and middle-SDI regions, combining tobacco control policies, including taxation and smoke-free legislation, with routine cancer screening programs could further reduce the incidence of urological cancers. For low-middle and low-SDI regions, priority should be given to strengthening primary healthcare infrastructure (e.g., establishing standardized cancer registries, improving access to histopathological diagnosis) and implementing community-based smoking cessation initiatives.
